# A novel mutation in retinitis pigmentosa GTPase regulator gene with a distinctive retinitis pigmentosa phenotype in a Chinese family

**Published:** 2010-08-15

**Authors:** Xunlun Sheng, Zili Li, Xinfang Zhang, Jing Wang, Hongwang Ren, Yanbo Sun, Ruihua Meng, Weining Rong, Wenjuan Zhuang

**Affiliations:** 1 Department of Ophthalmology, People Hospital of Ningxia Hui Autonomous Region, Yinchuan, China; 2 Department of Ophthalmology, Affiliated Hospital of Ningxia Medical University, Yinchuan, China; 3 Ningxia Medical University, Yinchuan, China; 4 Department of Ophthalmology, Affiliated Hospital of Medical College, Qingdao University, Qingdao, China; 5 Central Laboratory of Ningxia Medical University, Yinchuan, China; 6 Chongqing Medical University, Chongqing, China

## Abstract

**Purpose:**

To screen the mutation in the retinitis pigmentosa GTPase regulator (*RPGR) ORF15* in a large Chinese family with X-linked recessive retinitis pigmentosa and describe the phenotype in affected male and female carriers.

**Methods:**

Ophthalmic examination was performed on 77 family members to identify affected individuals and to characterize the disease phenotype. PCR and direct sequencing were used for screening mutations in the *RPGR* gene.

**Results:**

Mutation screening demonstrated a novel mutation *ORF15*+577_578 delAG, which caused an open reading frameshift and resulted in premature truncation of the RPGR protein. The mutation was detected in eight affected male individuals and 14 obligate female carriers of the family and was found to segregate with the phenotype in this family. The mutation led to a severe retinitis pigmentosa (RP) phenotype in male-affected individuals, with some variability in the age of onset of night blindness and visual acuity, but was recessive in female carriers without an RP phenotype. However, the state associated with the carrier was moderate to high myopia with the refractive error ranging from −5.00 D to 22.00 D in 14 female carriers.

**Conclusions:**

This novel mutation in *RPGR ORF15* causes a serious RP phenotype in males and no RP phenotype in female carriers. Moderate to high myopia was a particular feature for female carriers in this pedigree. Our finding expands the spectrum of *RPGR* mutations causing X-linked RP and expands phenotypic spectrum of the disease in a Chinese family. This finding will be useful for further genetic consultations and genetic diagnosis.

## Introduction

Retinitis pigmentosa (RP; OMIM 268000) is the most common form of inherited retinopathy and is characterized by loss of night vision in adolescence, side vision in young adulthood, and central vision in later life because of the progressive degeneration of rod and cone photoreceptor cells. The prevalence of RP was estimated to be one out of 4,000–5,000 individuals in Western countries [1] and one out of 3,784 individuals in China [2]. This disease affects approximately 1.5 million individuals worldwide [3].

RP shows considerable clinical and genetic heterogeneity, featuring wide variations in disease severity, clinical phenotype, age of onset, rate of progression, and mode of inheritance, and over 40 genes are involved. Despite intensive research on the pathogenesis of the disease, there is currently no effective treatment available.

Inheritance can be autosomal dominant, autosomal recessive, X linked, or in the rare cases taken as a digenic trait. However, in the majority of cases (about 50%–60% in Caucasians) it is impossible to establish the pattern of inheritance, and these cases are defined as “sporadic” [4]. The X-linked form (XLRP) accounts for 6%–17% of all RP cases, depending on the countries and the ethnic groups analyzed [5,6]. XLRP is the most severe type of retinal degeneration because of its early onset and has the most rapid progression [7]. Affected individuals experience a decrease in peripheral and night vision within the first or second decade of life and show partial or total blindness by the third or fourth decade [8]. Linkage analysis indicates six different loci have been clearly associated with the pathogenesis of XLRP, but most of the XLRP cases (80%–90%) are due to mutations in the RP guanosine triphosphatase regulator (*RPGR*; OMIM 312610) and the *RP2* (OMIM 312600) genes [9]. In total, approximately 70%–80% of XLRP cases carry mutations in *RPGR* and up to 20% in *RP2* [10].

The *RPGR* gene is located on chromosome Xp21.1 with 23 exons, including the tissue-specific alternatively spliced exons 9a, open reading frame 15 (*ORF15)*, 15a, and 15b [11–13]. The N-terminal part of RPGR contains a domain homologous to the regulator of chromosome condensation 1, the guanine–nucleotide exchange factor for the guanosine triphosphatase Ran that is important for the association with binding partners [14]. Although the *RPGR* gene has 23 exons, reports have indicated that pathogenic mutations cluster in exon *ORF15* [15,16]. These mutations include deletions, insertions, and substitutions and are associated with a wide variety of human clinical phenotypes [17–23]. It remains unknown how various mutations in *RPGR* can lead to significantly different clinical phenotypes.

The aim of this study was to screen the mutation in a large Chinese family with a possible XL inheritance pattern and to characterize the phenotypic manifestation associated with the mutation. The screening strategy for molecular genetics testing of XLRP cases is by direct sequencing and beginning with the screening of an XLRP male patient by *ORF15* mutation analysis, as proposed by Neidhardt and coworkers [14].

## Methods

### Study subjects

A family with a possible XL inheritance pattern was recruited at the Department of Ophthalmology in the People’s Hospital of the Ningxia Hui Autonomous Region from August 2007 to February 2009. There are 82 family members in the pedigree, and 77 members were successfully recruited. There were 41 males and 36 females and the age ranged from 4.5 to 76 years. All of them were healthy without other disease except three with hypertension. They received complete ophthalmic examinations, including routine ophthalmic examinations, Octopus perimetry, electroretinography (ERG), and color fundus photography. The individuals were diagnosed with nonsyndromic RP if they had a typical clinical history and features of RP, such as night blindness, decreased visual fields, and bone spicule pigmentation on fundus examinations. Syndromic RP, such as Usher’s syndrome, Leber congenital amaurosis, and Bardet–Biedl syndrome, were excluded. Control subjects included 80 healthy subjects (42 males and 38 females and the age ranged from 12 to 70 years) who had undergone detailed ocular examinations and were confirmed to be free of RP and other major eye diseases.

Written informed consents were obtained from all subjects and controls. The study protocol was approved by the Ethics Committee on Human Research, the People Hospital of Ningxia Hui Autonomous Region. All procedures in this study were performed in accordance with the Declaration of Helsinki.

### Mutational screening

Peripheral blood was obtained from 77 family members and 80 normal subjects. The blood were drawn with EDTA tubes and then stored at −20 °C in refrigerator for 1 to 7 days before processing. Genomic DNA was extracted using the QIAamp DNA Blood kit (Qiagen, Valencia, CA), according to the manufacturer’s instructions.

From the 200 µl input volume of EDTA-whole blood sample, a final 200 µl extracted volume was obtained. The steps were as follows: Samples were lysed and heated in the orbital shaker. Each lysate was transferred to a spin column in a rotor adaptor and if the lysate needed to be homogenized or cleared, it was transferred to the middle position of the rotor adaptor. Nucleic acids were bound to the silica membranes or purification resins of the spin column and washed to remove contaminants. The spin column was transferred to a collection tube for elution of purified nucleic acids. Four pairs of primers were designed according to the published exon *ORF15* of the *RPGR* gene sequence from GenBank (OMIM 312610; Table 1). Cycling conditions of the PCR were conducted for 34 cycles as follows: initial denaturation at 95 °C for 5 min, denaturation at 95 °C for 30 s, annealing at 57.5 °C for 45 s, elongation at 72 °C for 45 s, and a final single incubation at 72 °C for 5 min. A 5-μl aliquot of PCR product was subjected to agarose gel electrophoresis on a 2% agarose gel to confirm successful amplification before sequencing. The PCR products were then used for direct sequencing on a Perkin-Elmer 3100 Automated Sequencer (Applied Biosystems, Foster City, CA). The DNA sequences were compared with the human *RPGR* (ENSG00000156313) sequences in the Ensembl database. Detected sequence variants in the DNA sample were confirmed by bidirectional sequencing on another stock DNA sample.

**Table 1:** Primer sequences for polymerase chain reaction *RPGR ORF15* screening

**Primer**	**Primer sequence (5′→3′)**		**PCR product (bp)**
**Forward**	**Reverse**
ORF15 1	AGGAAGGAGCAGAGGATTCA	CCCTCTTCTTCCATTCTTCC	348
ORF15 2	GGGGAGAAAGACAAGGGTAG	TCCTTTCCCCTCCTCTACTT	444
ORF15 3	GGAAGAAGGAGACCAAGGAG	CCCATTTCCCTGTGTGTTAG	982
ORF15 4	GCAGGATGGAGAGGAGTACA	GAGAGAGGCCAAAATTTACCA	415

## Results

**Figure 1:**
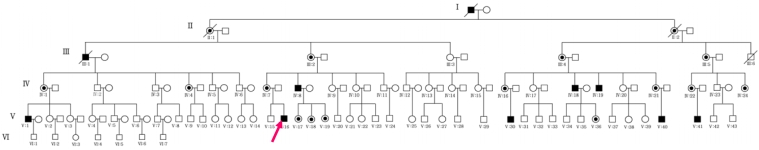
Pedigree with six generations. The circles represent females, and the squares represent males; slashed symbols indicate deceased family members. The filled black symbols denote family members with retinitis pigmentosa, open symbols indicate unaffected individuals, and circles with a dot indicate female carriers. The arrow marks the index patient (proband).

There are 82 family members in the pedigree, including five individuals who have passed away. Eight affected males were diagnosed by ophthalmic exam. A six-generation pedigree (with 41 males and 36 females still living) was compiled and revealed X-linked recessive inheritance (Figure 1). Eight affected males and 14 obligate carriers in the six-generation family were assessed.

### Male phenotype

**Figure 2:**
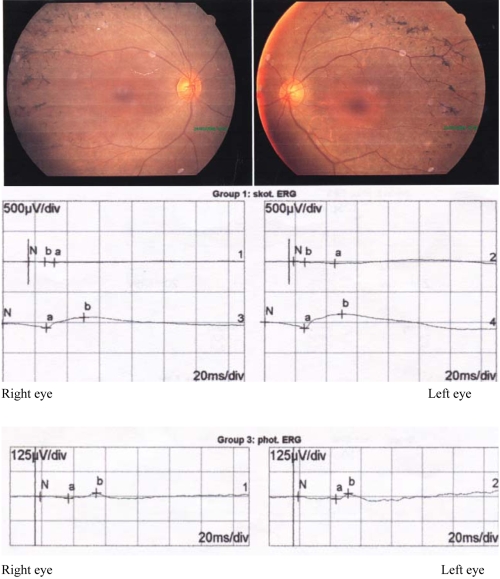
Fundus photographs and electroretinography results of the proband with X-linked retinitis pigmentosa,. The bone spicule pigmentation and attenuated retinal vessels can be seen bilaterally. Both the rod and cone electroretinogram amplitudes were below 10% of normal electroretinography (ERG; norms for scotopic [skot] ERG: b-wave 27.6 μV±5.2; norms for photopic [phot] ERG: b-wave 70 μV±8.9). Abbreviations: div means division.

The clinical data of eight affected males are summarized in Table 2. All affected males had mild myopia with refractive error ranging from −1.50 D to −3.00 D. The proband (V:16) was a 25-year-old man whose night blindness was noticed at the age of 2 by his mother, after which the diagnosis of RP was confirmed. He noticed blurred vision at age 13 but did not visit our hospital for examination until age 25. Visual acuity was 20/40 on the right eye and 20/60 on the left eye. Ophthalmologic examinations revealed pigmentation in the mid-peripheral fundi (Figure 2). Octopus perimetry showed tunnel vision and depression of threshold values. Both rod and cone electroretinogram amplitudes were below 10% of normal ERG (Figure 2). The proband’s uncle (IV:8) had onset in his early 20s, and bilateral legal blindness was noted in his early 40s. One of the participants (V:1), who had onset at age 27, was found to have normal central vision and 20° visual fields at age 33. The two youngest affected males, a 4.5-year old and an 8-year old (V:30, V:41), were asymptomatic with mild retinal changes. The other 33 males without the mutation were hyperopic or low to moderately myopic with the median spherical equivalent of –1.75 D (range, +3.50 D to –3.75 D)

**Table 2:** Summary of clinical findings of affected males in the family with X-linked retinitis pigmentosa.

**Family member/age (Y)**	**Onset**	**Optics**	**BCVA**	**Fundus**	**VF**	**Rod** **ERG**	**Cone** **ERG**
IV:8/54	20	−1.50/-3.00	20/200 20/200	Bilateral extensive chorioretinal atrophy and midperipheral pigmentation	ND	Extinguished	Extinguished
IV:18/52	21	−2.50/-1.75	20/80 20/60	Bilateral extensive chorioretinal atrophy and midperipheral pigmentation	TV	Extinguished	Extinguished
IV:19/46	24	−0.75/-1.25	20/80 20/80	Bilateral extensive chorioretinal atrophy and midperipheral pigmentation	TV	Reduced	Reduced
V: 1/33	27	−0.25/-0.50	20/20 20/20	Few periphera pigmentation	20 degrees	Reduced	Normal
V: 16/25	2	−1.75/-2.25	20/40 20/60	Bilateral extensive chorioretinal atrophy and midperipheral pigmentation	TV	Seriously reduced	Seriously reduced
V: 30/8	AS	+1.00/+1.50	20/20 20/20	Few periphera pigmentation	ND	ND	ND
V: 40/11	10	−2.00/-2.75	20/20 20/20	Few periphera pigmentation	ND	Reduced	Normal
V: 41/4.5	AS	+1.00/-0.50	20/20 20/20	Few periphera pigmentation	ND	ND	ND

Abbreviations: AS, asymptomatic; BCVA, best-corrected visual acuity; VF, visual field; ND, not determined; TV, tunnel vision.; ERG, electroretinography.

### Female phenotype

The clinical data of 14 female carriers are summarized in Table 3. All female carriers in this pedigree showed moderate to high myopia with a refractive error ranging from −5.00 D to −22.00 D. The proband’s mother (IV:7), a 56-year-old heterozygous woman, did not have night blindness. She had high myopia with a best corrected visual acuity of 20/20 oculus uterque (OU). The fundus examination showed chorioretinal thinning, myopic optic discs, and peripapillary atrophy, which were consistent with high myopia. No spicule formations were observed in the peripheral fundi. The ERG showed a normal response.

**Table 3:** Summary of clinical findings of female carriers in the family with x-linked retinitis pigmentosa.

**Family member/age (Y)**	**BCVA**	**Optics**	**Fundus**	**VF**	**Rod ERG**	**Cone ERG**
III:2/76*	20/100;20/100	−8.50/-8.00	Bilateral myopic fundi	NA	NA	NA
III:4/ 74*	20/80;20/60	-10.25/-9.75	Bilateral myopic fundi	NA	NA	NA
III:5/ 70*	20/60;20/50	−8.75/-6.25	Bilateral myopic fundi	NA	NA	NA
IV: 1/ 57	20/20;20/20	−6.25/-6.50	Normal	Full	Normal	Normal
IV:4/48	20/50;20/60	−6.75/-7.25	Slightly bilateral Chorioretinal atrophy	Full	Normal	Normal
IV: 7/ 56	20/20;20/20	−6.75/-6.25	Bilateral myopic fundi	Full	Normal	Normal
IV: 16/ 38	20/25; 20/30	-10.75/-10.25	Bilateral myopic fundi	Slightly reduced	Normal	Normal
IV:2 1/ 34	20/20; 20/20	−5.00/-6.25	Slightly bilateral Chorioretinal atrophy	Full	Normal	Normal
IV:2 4/ 28	20/20; 20/20	−6.00/-8.25	Normal	Full	Normal	Normal
IV: 22/ 28	20/20; 20/20	−8.00/-8.25	Bilateral myopic fundi	Full	Normal	Normal
V: 17/27	20/50;20/40	−15.00/-12.00	Bilateral myopic fundi	Reduced	Reduced	Reduced
V: 18/25	20/22;20/20	−5.50/-6.75	Normal	Full	Normal	Normal
V: 19/23	20/30;20/60	−18.00/-22.00	Bilateral myopic fundi	Reduced	Reduced	Reduced
V: 36/ 29	20/20; 20/20	-10.0/-9.25	Bilateral myopic fundi	Slightly reduced	Normal	Normal

Abbreviations: BCVA, best-corrected visual acuity; VF, visual field; NA, not available; ERG, electroretinography; * cataract

The proband’s uncle (IV:8), who had severe RP and mild myopia, has three daughters, all of whom were heterozygotous carriers (V:17, V:18, V:19) and exhibited moderate to high myopia. One daughter, aged 23 wearing glasses right (R) −18.00 D, left (L) −22.00 D, had 20/30 and 20/60 central vision in both eyes. Deteriorated central visual acuity was noted at age 9 due to high myopia. A fundus examination revealed the retinal and optic disc changes that were consistent with high myopia. No spicule formations were observed in the peripheral fundi. ERG scotopic amplitudes were 75% of the normal, and photopic amplitudes were 90% of the normal. One of her older carrier sisters, wearing glasses R −15.00 D, L −12.00 D, had 20/50 and 20/40 central vision (right and left eye, respectively) with the same retinal change as mentioned above. The other older carrier sister, wearing R −5.50 D, L −6.75 D, showed normal central vision and a normal retinal appearance. Among 22 females without the mutation, 20 had a median of refractive error of –2.50 D (range, 2.75 D to –4.50 D) and two were found to have high myopia with a refractive error ranging from −6.25 D to 8.75 D.

### Mutational analysis

Among the 77 family members and 80 control subjects, a total of three sequence changes in the *RPGR ORF15* were identified, including one novel deletion change (Table 4). One variant (N547N) was a synonymous change, and one (I559V) was a missense change. Both N547N (rs12687163) and I559V (rs12688514) are common single nucleotide polymorphisms (SNPs) registered in the dbSNP database.

**Table 4:** Sequence variations detected in the exon *ORF15* of *RPGR* gene among Chinese RP patients and control subjects

** **	** **	** **	** **	**Variation frequency**			
**Location**	**Nucleotide change**	**Residual change**	**Description**	**Patients n=8**	**Carriers n=14**	**Normal n=55**	**Controls n=80**
ORF15	g.ORF15+1677A>G (c.3430A>G)	p.I559V (p.I1144V)	rs12688514	4	6	22	36
ORF15	g.ORF15+1643T>C (c.3396T>C)	p.N547N (p.N1132N)	rs12687163	3	5	16	32
ORF15	g.ORF15+577_578delAG (c.2330_2331 delAG)	Lys192fsTer248 (p.Lys777fsTer833)	Novel	8	14	0	0

**Figure 3:**
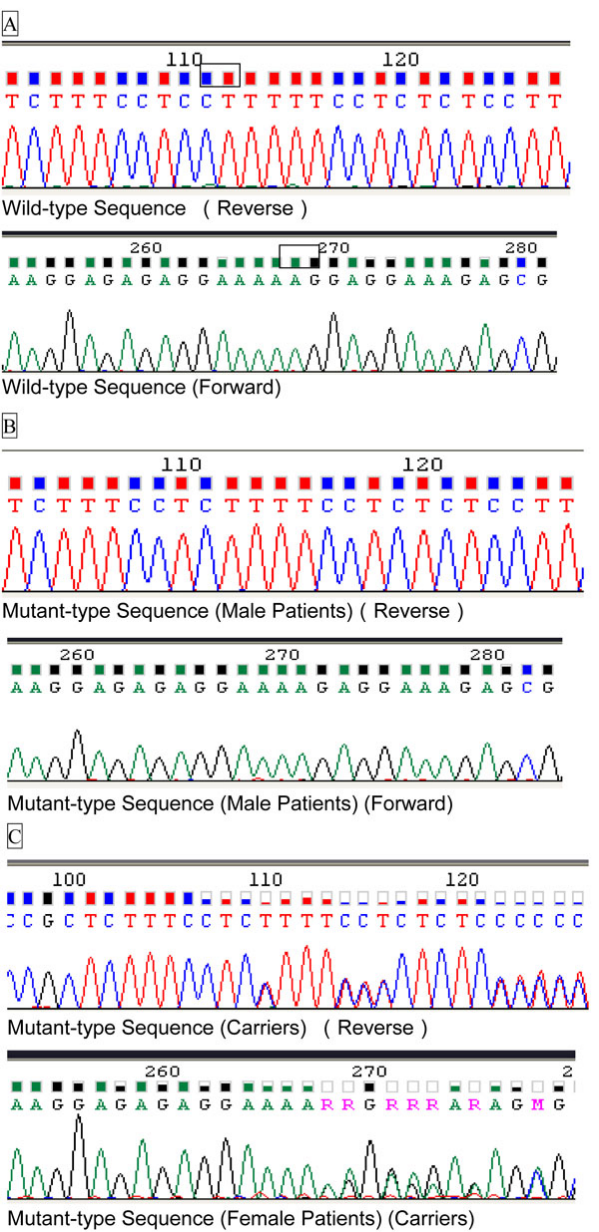
Results of nucleotide sequencing analysis. **A**: Family has a ORF15+577_578delAG mutation. The upper sequences (**A**) are the normal alleles. The middle sequences (**B**) are homozygous mutation identified in affected male and the lower sequences (**C**) are heterozygous mutation identified in obligate female. The black rectangles indicate the deleted two nucleotides. The filled squares indicate homozygous condition of the base. The partially filled squares indicate heterogeneous condition of the base.

A 2-bp deletion at positions 577 and 578 in exon *ORF15* (Figure 3) was identified in affected males and obligate female carriers; this deletion is predicted to create an early termination at codon 248 (Lys192fsTer248). *ORF15* Lys 192fsTer248 refers to a frameshift mutation in which Lys 192 is the first amino acid altered and the termination of the *ORF* is at residue 248. The mutation results in a premature stop codon predicted to prematurely truncate the RPGR protein. The homozygous mutation was identified in all affected males, and the heterozygous mutation was identified in all obligate female carriers. The mutation co-segregated with disease in eight affected males and 14 female carriers in the family and was not observed in any of the unaffected family members or the group of normal controls.

## Discussion

To date, approximately 70%–80% of XLRP cases are found to carry mutations in *RPGR* and up to 20% are found to carry mutations in *RP2* [10]. The exon *ORF15*, which contains a purine-rich domain of an approximately 1706-bp coding sequence and is predicted to encode a repetitive glycine and glutamate region at the C-terminus of the protein, is a mutation hot spot for XLRP [12]. Between 30% and 80% of *RPGR* mutations are identified in exon *ORF15*, followed by mutation frequencies that are similar for *RP2* and *RPGR* exons 1–15 [11–13,24–28]. No mutation has yet been identified in exons 16–19. Based on these results and to keep costs low, Neidhardt and coworkers proposed a screening strategy for routine molecular genetics testing of XLRP cases by direct sequencing and recommended beginning with the screening of an XLRP male patient by *ORF15* mutation analysis [14]. After identification of an *ORF15* mutation, the molecular diagnosis is considered confirmed since patients with an additional mutation in exons 1 through 15 of *RPGR* and *RP2* have not been reported. If no *ORF15* mutation is identified, Neidhardt and coworkers recommended to continue screening of mutations with *RP2* rather than *RPGR* because *RP2* is composed of only five exons, whereas 15 exons need to be screened to complete the sequence analysis of *RPGR* without any clear benefit in terms of likelihood to detect the causative mutation. Accordingly, we began our screening on the proband in an X-Linked recessive RP family by *ORF15* mutation analysis. After an *ORF15*+577_578delAG mutation was identified, direct DNA sequence analysis was performed for all family members and 80 controls. The segregation of the *ORF15*+577_578delAG mutation within the family was verified by finding it was only present in all affected male individuals and 14 female carriers of the family but was not present in other normal family members. In addition, population frequency analysis showed the *ORF15*+577_578delAG mutation was absent in 80 healthy controls. *ORF15*+577_578delAG mutation causes an open reading frameshift, which is predicted to result in a stop codon at position 248 leading to a premature termination and to a truncation of 319 amino acids from the protein. Our results showed that the *ORF15*+577_578delAG mutation was involved in the RP pathogenesis in this Chinese family. We confirmed that our approach to amplify the complete exon *ORF15* in a single PCR reduces costs and labor.

To date, more than 100 different mutations in exon *ORF15* have been reported [23,29]; however, the precise role of *ORF15* is still unclear. Studying the genotype–phenotype correlation can elucidate the expressivity and penetration of the phenotype in a patient with a specific mutation in *RPGR* [20].

Earlier reports have suggested that there is significant interfamilial variability in the XLRP phenotype [30] and different *RPGR* mutations can cause divergent retinitis pigmentosa phenotypes [22,30]. However, within a given family the phenotype is usually homogeneous [31,32], but intrafamilial phenotypic heterogeneity has also been reported [19,21,33]. In this study we further described this intrafamilial phenotypic heterogeneity. We showed that a frameshift mutation in *ORF15* of *RPGR* causes a severe XLRP phenotype; however, the male phenotype is distinctly different. The finding of a single mutation causing different phenotypes suggests the presence of modifiers, which may be either genetic, environmental, or both [23].

Historically, most *RPGR* mutations were considered to be recessive with clinically normal female carriers or mild phenotypes [34–38]. However, severely affected carriers have been reported [34,39]. For *RPGR*, the high penetrance in females seems to be most common with truncating mutations particularly in exon *ORF15* [15,16]. Neidhardt and coworkers identified three pathogenic sequence alterations in the mutational hot spot exon *ORF15* that also lead to disease expression in female carriers [14]. The mutation c.2548delG is found in a family in which the mother, in addition to three of her sons, is affected [14]. The same mutation occurs in a second family with reduced disease expression in female carriers [14].

What is intriguing is the fact that the truncating mutation in the exon *ORF15* identified here causes a serious RP phenotype in males and no RP phenotype in heterozygote female carriers. All affected males exhibited RP but all female carriers were recessive with RP in the same family with the same mutation. The state associated with the carrier was moderate to high myopia with a refractive error ranging from −5.00 D to 22.00 D in 14 female carriers. However, the *ORF15*+577_578delAG mutation was not segregated with high myopia because high myopia was also observed in two female family members without the mutation. Therefore, the findings of our study do necessarily signify that moderate to high myopia is more prevalent in females in this family with an *ORF15*+577_578delAG mutation, and it is conceivable that high myopia is also a variable expression of this *RPGR* mutation. Other studies have found high myopia in patients with XLRP, particularly in females with *RPGR* mutations. Yang and coworkers identified a GA deletion at *ORF15*+483_484delGA of *RPGR* in a family with XLRP in which all female patients had a refractive error of −6 D or more [23]. Koenekoop and coworkers identified two *RPGR* mutations in two families with XLRP [19]. A Glu 414 (2-bp deletion) frameshift mutation was found in nine affected males and six affected females in family I in which all affected females had myopia with a refractive error ranging from −2 D to −9 D and all affected males were emmetropic. An intervening sequence (IVS) 2–1 (G to A) splice site mutation occurred in one affected male and two affected females in family II in which all affected individuals had high myopia with a refractive error ranging from −7 D to −10 D. Jin and coworkers identified a mutation, *ORF15*+1232_ 1233delGG, in a family with XLRP. Among six recruited individuals, two affected females had high myopia with a refractive error ranging from −14.50 D to 18.50 D, two affected males had low to moderate myopia with a refractive error ranging from −1.000 D to 5.00 D, and two unaffected individuals were emmetropic [20].

Among the *RPGR ORF15* sequence variants detected in our Chinese subjects, the two previously reported (rs12687163 and rs12688514) SNPs were found not only in patients but also in normal family members and control individuals and could be excluded as disease-causing mutations.

In conclusion, we identified a novel mutation, *ORF15*+577_578delAG, in the *RPGR* gene in a large Chinese family with X-linked recessive retinitis pigmentosa and documented the clinical manifestations. Our review of the phenotypes by genotype analysis showed that there is significant intrafamilial variability in the XLRP phenotype. Moderate to high myopia is a particular feature for female carriers in this pedigree. This feature may be the most reliable guide to the female carrier status in XLRP, its presence implying a mutation in the *RPGR* gene. Our finding expands the spectrum of *RPGR* mutations causing XLRP and the phenotypic spectrum of the disease in a Chinese family, and will be useful for additional genetic consultation and genetic diagnosis. Further investigations will identify and characterize the possible genetic modifiers that determine the clinical outcome of *RPGR* mutations in female carriers.
